# Supplementary material for: Are carbon emissions trading and green financial instruments synergistic? -Comprehensive quantitative research based on content analysis
Source: PLoS One. 2024 Mar 7;19(3):e0298601. doi: 10.1371/journal.pone.0298601 (PMC10919601; doi:10.1371/journal.pone.0298601)
Supplement: S1 Data — (ZIP) [file pone.0298601.s001.zip › Data/地级市Z_F工作报告.zip!/┴╔─■/░░╔╜╩╨/└┤╫╘╣1⁄2╓┌║┼í╛┬φ┐╦╩2╛▌═°í┐_2016.docx]

各位代表：

现在，我代表市政府向大会报告工作，请予审议，并请市政协各位委员提出意见和建议。

一、“十二五”时期及2015年工作回顾

“十二五”时期是鞍山发展不平凡的五年。五年来，市政府深入贯彻党的十八大和十八届三中、四中、五中全会精神，在省委、省政府和市委的正确领导下，在市人大、市政协监督支持下，积极抢抓新一轮东北振兴机遇，主动适应经济发展新常态，全市经济社会保持平稳健康发展。

――经济实力总体提升。全市地区生产总值年均增长7.5%，人均地区生产总值超过1万美元，公共财政预算收入年均下降6.5%，社会消费品零售总额年均增长13.5%，城镇登记失业率控制在3%以内，城乡居民人均可支配收入分别年均增长11%和12.9%。

――改革开放全面深入。五年来，我们始终坚持以改革破难题，以开放促发展。在全省率先建立行政权力正面清单和权责清单，实行“三证合一”、“三台合一”。市级行政权力由5511项减少到1462项，精简比例73.5%。累计下放市级管理权限200余项。成立公共资源交易中心，新建公共行政服务大厅。开发园区体制机制全面创新。政府机构改革、事业单位分类改革、国资国企改革、农村综合改革、社会事业改革等领域取得积极进展。对外开放领域和深度逐步拓展，预计“十二五”期间，实际直接利用外资54.5亿美元，累计完成出口129亿美元，分别比“十一五”增长98.8%和22.5%。

――创新能力显著增强。五年来，我们把创新摆在与改革开放同等重要位置，积极构建创新服务平台，创新引领作用更加突出。累计创建产业技术创新战略联盟11个，省级以上企业研发机构101家。发明专利申请量、授权量实现成倍增长，7项专利获得中国专利优秀奖，6个项目获得国家科技进步奖。全市高新技术企业发展到119户，比2010年末翻一番。我市成为全省唯一荣膺“全国科技进步先进市”五连冠城市。

――结构调整继续推进。五年来，我们变压力为动力、化被动为主动，不失时机加快结构调整。三次产业比重由2010年的4.4:54.3:41.3调整到5.7:47:47.3。鞍钢集团进入世界500强，鞍钢船板、铁路钢轨等研发技术达到国际领先水平。民营经济占全市地区生产总值超过70%。与2010年相比，钢铁、菱镁深加工率分别提高10个百分点，装备制造业比重提高6个百分点，新兴产业占比达到8.7%。14个服务业集聚区主营业务收入年均增长16%，现代服务业增加值年均增长超过20%。粮食综合生产能力稳定在130万吨以上，五年连获丰收；农机作业服务面积达到180万亩，综合机械化水平达到78%。县域工业园区入驻企业超过500家。海城市蝉联中国中小城市科学发展综合实力百强县前十强。

――城乡环境加速改善。五年来，我们统筹推进城乡建设，着力改善生态环境。累计建成兴业大道、四达大道、通海大道、鞍山大道、建国大道等城市主次干道452条，新增公路473公里。新修农村道路1984公里，在全省率先实现全部行政村通柏油路、通客车。鞍山机场开通北上广航线。累计投资134亿元，持续推进“蓝天、青山、碧水、净土”生态环境保护工程和“绿化、亮化、净化、美化”城乡环境治理工程。完成大气污染治理项目240项，矿山生态治理面积3.27万亩，城区7座污水处理厂全部建成使用，城市污水处理率由不足50%提升到94%，生活垃圾无害化处理率、覆盖率均实现100%。城市森林覆盖率提高到47.9%，我市被评为国家森林城市、国家园林城市和全省青山工程示范市。

――民生保障不断强化。五年来，我们把增进民生福祉作为根本目的，持续实施惠民工程。累计投入100多亿元，办理惠民实事140余件。年均实名制就业10万人次以上，被评为全国创业先进城市。在全省率先开展新型农村合作医疗和城镇居民社会养老保险。城乡居民最低生活保障标准分别提高66.7%和84.2%。累计建设回迁房731.8万平方米，回迁居民8.1万户。教育标准化程度持续提升。职业教育改革发展走在全国前列。第一轮国家医药卫生体制改革试点任务全面完成。市奥体中心建成使用。成功承办第十二届全运会鞍山赛区四项赛事。蝉联全国双拥模范城市五连冠。全国文明城市创建工作取得显著成效。

各位代表，回顾刚刚过去的2015年，我市经济发展遇到了严峻的挑战，但是我们迎难而上，攻坚克难，各项工作取得了积极效果，大部分经济指标增速高于全省平均水平。预计，全年实现地区生产总值2326亿元，按照可比口径增长3.5%；公共财政预算收入128.8亿元，按照可比口径下降4.4%；社会消费品零售总额970亿元，增长8.1%；城镇登记失业率控制在3%以内；城镇常住居民人均可支配收入29943元，增长7.5%，农村常住居民人均可支配收入13117元，增长8.5%；万元地区生产总值综合能耗降低4.5%。一年来，我们主要做了以下几方面工作：

（一）保持经济运行平稳

召开全市企业家和处级以上干部服务企业两个“千人大会”，汇编、出台扶持企业发展政策153条。深入实施市级领导服务企业包保项目责任制，帮助企业解决各类难题400多个。积极支持鞍钢科技研发和产品创新，鞍钢60万吨高强度汽车板项目投入运营。全年鞍钢科技创新产品产量超过500万吨，新产品比例达到25.5%。

开展招商引资和腾迁净地两个“百日行动”，先后赴日本、新加坡和京津冀、长三角、珠三角等国家地区开展主题招商，签约项目275个、落地项目196个；腾迁净地43宗、276万平方米。全市在建亿元以上项目668个。

认真贯彻党中央、国务院支持东北振兴重大举措。我市荣获国家循环经济示范市、绿色转型发展示范市和“宽带中国”示范市。经济开发区获批国家循环化改造示范试点园区，汤岗子新城列入国家循环经济示范区，立山区列入国家老工业区搬迁改造试点，大孤山工矿区列入国家独立工矿区搬迁改造试点，累计争取上级资金27.9亿元。

（二）转型升级步伐加快

新增省级产业技术创新战略联盟6家、省级产业专业技术创新平台4个、院士专家工作站5个。全市新增高新技术企业24家，增长19%；高新企业营业总收入达到147.8亿元，增长13%。

制定《鞍山制造2025》和《工业绿色转型发展实施方案》。优势产业进一步改造提升，新兴产业不断壮大。紫竹集团100万吨城市轨道钢项目建成投产，荣信集团1000兆瓦柔性直流输变电装备研制成功，激光产业园荣获国家特色产业基地。轻工业加速发展，维达纸业二期建成投产，朗致药业基地开工建设。

深入实施服务业四年行动计划，8个大型服务业项目竣工营业。新增5家金融机构，44户企业登陆多层次资本市场。新增金融机构本外币贷款260亿元，增长14.3%。全市电子商务交易额突破400亿元，增长33%。新分立生产性服务业企业50户。新增中介服务业企业300户。千山风景区列入国家5A级景区预备名录，玉佛苑更名为玉佛寺，成功举办中国数字娱乐电子竞技大赛，全年旅游综合收入增长15%。预计全年完成商品房销售额148.9亿元，销售面积322万平方米。

全年粮食总产量实现141万吨。海台岫三个农高区入园项目71个，全年开工建设亿元以上农产品深加工项目22个，台安安井10万吨速冻食品深加工一期建成投产。新培育规模以上农事龙头企业10家，农民专业合作社发展到2702个,家庭农场发展到252个。鞍山南果梨、岫岩滑子蘑、鞍山君子兰被评为国家地理标志保护产品。

（三）各项改革不断推进

新取消行政权力51项，下放36项，扎实做好下放权力的跟踪与监督考评。企业投资项目审批流程由5个阶段整合为3个阶段，审批时限从54个工作日压缩至34个工作日。不断优化公共资源交易流程，政府采购和土地交易办理时限缩短三分之一。我市成为全省企业投资项目管理体制改革试点、投资项目在线审批监管平台试点和负面清单改革试点。

国有资产监督管理体制不断完善，出台《进一步深化全市国企改革的意见》及相关配套文件，完成73户国有企业与原主管部门脱钩。商事制度改革加速深化，实行“一照一码”登记制度，新设立市场主体3万户，新增注册资本271亿元。财税体制改革不断推进，全面清理财政沉淀资金，统筹用于促进经济发展和保障民生支出。全面完成食品药品监督管理体制改革，基层食品监管能力大幅提高。农村改革步伐加快，完成61万亩农村土地确权登记颁证工作，海城获批农村集体经营性建设用地入市等3项国家级改革试点。

出台《鞍山市投资、贸易、金融、商务便利化实施意见》。启动一次申报、一次查验、一次放行的通关便利化模式。海城西柳被确定为首批省级重点培育内外贸结合商品市场试点。

（四）社会事业全面发展

为城乡群众办的20件实事全面落实。争取棚户区改造资金38.6亿元，在建回迁房项目18个，其中竣工项目11个，建筑面积133万平方米，安置回迁居民1.9万户。全年城镇实名制就业8.5万人，保持“零就业家庭”动态为零。城乡居民最低生活保障标准分别提高7%和10%。企业退休职工养老金提高199元，城乡居民养老保险基础养老金上调至85元。新型农村合作医疗筹资标准提高到500元。困难家庭大学生就学资助标准提高30%。完成阶梯气价调整，下调居民采暖费。

全面完成数字鞍山地理空间框架建设和城市地下管线普查工作。兴业大道、千山南路、辽河路一期建成通车。鞍海路等25条道路改造完成。鞍山路等29项供水配套完成。维修改造供暖锅炉91台、供暖管网97公里。新建、改造城市公交候车亭676组，农村公路候车亭64个。新增农村饮水安全工程97处。完成鞍山机场航站楼扩建，全年进出港旅客突破12万人次。鞍海一体化进程加快，汤岗子新城建成“五横十纵”主干路网，中央活力区路网全面贯通。

编制实施《城市环境总体规划》。蓝天工程完成鞍山二热电关停、鞍钢二发电1号机组脱硝、宝得2台烧结机淘汰和中骏锅炉房淘汰等7个项目，拆除锅炉53台，购置新能源公交车200辆、“油改气”100辆，更新改造出租车1211辆。青山工程完成荒山荒地造林3.8万亩，封山育林1.2万亩，矿山生态环境治理3759亩。碧水工程完成永宁污水处理厂和台安经济开发区污水处理厂建设。宜居乡村建设投入资金2亿元，完成清理路边垃圾等综合治理工程。

新建改造15所幼儿园、20所中小学和4所高中，师范学院新校区开工建设，市职教城获批辽宁省现代职业教育实验区。顺利通过国家卫生城市复检初审。在全省率先启动新一轮医药卫生体制改革，实施分级诊疗，全地区公立医院实行药品零差价销售。市公共卫生中心等项目加快建设。开工建设市级养老院。完成市博物馆升级改造。新增社区书屋148个、图书馆分馆和少儿图书馆分馆16个。成功举办鞍山市第十届运动会。双拥优抚安置服务体系不断丰富，圆满完成第八轮双拥模范城考评工作，民兵预备役建设取得显著成绩。全面开展创城攻坚，实施环境卫生、交通安全、营运秩序等专项整治。完善立体化社会治安防控体系建设，严厉打击各类违法犯罪行为。全年未发生重特大生产安全事故和食品药品安全事件，社会保持和谐稳定。外事、侨务、对台、残联、统计、接待、史志、档案、老龄、气象、地震、人防等工作取得了新成绩。

（五）政府自身建设不断加强

深入学习习近平总书记系列重要讲话精神，扎实开展“三严三实”专题教育，认真贯彻省委提出的“八个带头、八个自觉”要求。全年办理人大代表建议和政协提案583件，见面率、办复率均为100%。认真办理市人大关于加强水资源管理“一号议案”，实行最严格水资源管理制度。制定出台《市政府关于全面落实党建工作和党风廉政建设责任制的规定》，带头执行“一岗双责”责任制。全面整治各级政府机关不作为、乱作为等突出问题，机关作风不断改进。

各位代表，“十二五”时期取得的成绩来之不易。这些成绩是省委、省政府和市委正确领导的结果，是市人大、市政协有效监督、大力支持的结果，是全市广大干部负重前行、奋力拼搏的结果，是全市人民共同努力的结果。在此，我代表市政府，向全市人民，向各位代表和委员，向各民主党派、工商联、无党派人士和各人民团体，表示诚挚的感谢！向参与鞍山发展建设的中省直单位、驻鞍部队、武警官兵，向所有关心支持鞍山发展的老领导、老同志，向所有为鞍山发展做出贡献的海内外朋友，表示衷心的感谢！

在看到成绩的同时，我们也清醒地认识到鞍山的发展还面临着一些困难和挑战。主要表现在：传统产业转型升级难度加大，新的经济增长点尚未形成有效支撑；财政收支矛盾凸显，政府债务负担较重；制约经济发展方式转变的体制机制障碍依然突出，市场活力还需进一步释放；营商环境仍需改善，服务意识、诚信意识还需进一步增强；创新创业的氛围不够浓厚，企业创新能力不强，大项目、好项目不足；一些历史遗留问题尚未解决，民生改善仍有短板；一些干部对新常态的认识还不到位，引领新常态的本领还需提高。

尽管我市大部分经济指标增幅高于全省平均水平，但有些指标还是没有完成预期目标。原因是多方面的：从经济因素上看，我市原材料和重工业比重偏高，受外部环境影响较大，钢材等主导产品价格持续下跌，导致部分行业和企业生产经营困难。鞍钢集团遇到了前所未有的困难，生产经营受到较大影响。加之我们利用市场机制，淘汰落后产能，压减过剩产能，减少无效投资，规模以上企业比上年减少，相应拉低了经济增长幅度。从政策因素上看，我们全面落实结构性减税和普遍性降费政策，通过实施“营改增”政策，落实支持中小微企业税收优惠政策，取消并减免缓征行政事业性收费和政府性基金项目，这些政策性因素下拉了财政收入增幅。从自身主动作为因素上看，我们贯彻省委、省政府部署，按照“三严三实”要求，依法依规、严格做实经济指标，降低了指标增幅。虽然主要经济指标增速与预期目标存在差距，但全市各级领导干部为经济的平稳增长也付出了艰苦的努力。

二、“十三五”时期总体要求和目标任务

“十三五”时期是我市创新发展的攻坚期、协调发展的关键期、绿色发展的转型期、开放发展的拓展期、共享发展的决战期。我们必须全面分析经济形势，把握经济发展趋势，明确发展方向。一是必须主动适应经济发展新常态。认识新常态、适应新常态、引领新常态，是做好当前和今后一个时期经济工作的大前提、大逻辑。必须看到，经济发展正在由规模速度型粗放增长转向质量效率型集约增长，由增量扩能为主转向调整存量、做优增量并存的深度调整，由传统要素投入转向创新驱动和消费拉动。我们要加快转变经济发展方式、调整经济结构，培育形成新的增长动力。我们要把握关键点，找准发力点，瞄准突破点，采取有效措施，精准发力，精准施策。二是必须牢固树立“五大发展”新理念。坚持创新发展，把创新贯穿政府一切工作；坚持协调发展，提高经济社会发展整体性；坚持绿色发展，推进美丽鞍山建设；坚持开放发展，提高开放型经济水平；坚持共享发展，让全市人民共享发展成果。三是必须全面落实“四个着力”新要求。着力完善体制机制，突出深化改革治本之策；着力推进结构调整，抓住结构优化关键之举；着力鼓励创新创业，把握创新驱动决胜之要；着力保障和改善民生，筑牢民生改善稳定之基。四是必须有效激活“四个驱动”新引擎。坚持以创新驱动打造新的生产力，坚持以改革驱动激发内生动力，坚持以市场驱动增强发展活力，坚持以开放驱动培育国际竞争力。五是必须培育壮大“六个新增长点”。加快传统产业转型升级，培育壮大新兴产业，集中力量发展现代服务业，加速推进新型城镇化，切实转变农业发展方式，激发民营经济发展活力。

“十三五”时期主要目标是：地区生产总值年均增长7%左右，公共财政预算收入年均增长5%以上，社会消费品零售总额年均增长8%以上，城镇登记失业率控制在3.5%以内，城乡居民收入增长与经济发展同步。努力保持在全面建成小康社会实践中稳中求进，保持在全省老工业基地振兴发展中走在前列，保持在东部地区工作实绩考核中居于领先。

“十三五”时期，我们将下大力气重点抓好五项工作。一是着力优化经济结构。加速推进传统产业改造升级，到2020年，力争全市60%以上的传统企业迈向中高端水平；加快发展先进制造业，新兴产业占规模以上工业比重达到15%以上，高新技术产业增加值占地区生产总值比重20%以上，研发经费投入占地区生产总值比重提高到2%以上；服务业占地区生产总值比重提高到49%以上。二是着力推进一批重大项目。围绕新一轮东北振兴政策，争取一批国家和省重点支持项目；围绕国内外500强和行业领军企业，吸引一批具有牵动性的大项目；围绕先进地区产业转移，引进一批促进结构调整的重点项目；加快推进一批重大项目投产达产。三是着力实施新型城镇化。坚持规划引领、以人为本、有序推进。进一步完善城市功能，优化城市环境，加强生态保护，加快宜居城市、智慧城市建设，到2020年，全市户籍人口城镇化率提高到75%以上，有序推进农村劳动力向城镇集中。四是着力改善民生。到2018年末，完成81个贫困村、2.68万贫困人口的脱贫攻坚任务，摘掉岫岩省级扶贫开发重点县“帽子”。全面落实低收入群众增收行动计划，确保实现城乡居民收入倍增，形成共建共享的包容性发展新机制，增强人民群众的幸福感、获得感。五是着力改善营商环境。全面建设法治政府和诚信政府，全力推进“诚信鞍山”建设，营造亲商、安商、富商的环境和激励大众创业、万众创新的氛围。

三、2016年重点工作任务

2016年是“十三五”规划开局之年，是推进结构性改革攻坚之年，做好全年工作对实现全面建成小康社会目标至关重要。我们要深入贯彻中央和省经济工作会议精神，按照市委十一届十次全会部署和“十三五”规划的目标要求，努力实现地区生产总值增长5.5%左右，固定资产投资增长6%左右，社会消费品零售总额增长8%左右，外贸出口额增长5%左右，公共财政预算收入增长3%左右，规模以上工业增加值增长3%左右，城镇登记失业率控制在3.5%以内，城乡居民收入增长与经济发展同步，努力保持在全面建成小康社会实践中稳中求进，保持在全省老工业基地振兴发展中走在前列，保持在东部地区工作实绩考核中居于领先，实现“十三五”开好局起好步。重点做好以下九个方面工作：

（一）全力保持经济平稳增长

工业是我市经济的基础，稳增长关键是稳工业。实施降低企业成本专项行动，减轻企业税费负担。制定扶持政策，增加企业优质产品本地化协作配套，扩大企业市场份额。鼓励企业开发适销对路的新产品，开展个性化定制、柔性化生产。继续实施市级领导服务企业责任制，帮助企业解决实际困难，支持企业早日走出困境。

发挥有效投资对稳增长调结构的关键作用。全面对接国家新一轮东北振兴政策，加快推进齐大山工矿区搬迁改造纳入国家试点。积极争取国家基础设施建设、棚户区改造、生态环保等重大项目。加大招商引资力度，创新招商方式，开展务实招商、精准招商，注重项目的质量效益和开工率。拓宽招商区域和渠道，强化招商引资跟进机制，实行项目建设动态管理，严格项目考核机制。确保全年开工项目1000个，其中亿元以上项目426个。

扩大消费供给，不断增强消费拉动经济的基础作用。培育新型消费市场，促进信息消费、旅游消费、健康消费、休闲消费等多元化消费模式。加快推进养老、养生、教育、文化、体育等新型服务业态发展。落实“互联网+”行动计划，推进实体店网上商城建设，实现线上线下融合发展。推进兴隆大家庭、苏宁生活广场、义乌中国小商品城一期、戴维德水上乐园、汤石幸福小镇、东泰盛唐水世界等项目竣工营业。

主动融入国家“一带一路”和京津冀协同发展战略，千方百计扩大出口。落实出口信贷、出口退税、通关一体化和跨境电子商务等方面政策，降低企业进入国际市场的门槛、成本和风险。深入实施《外贸出口五年倍增计划》，积极培育外贸出口龙头企业，优化出口产品结构，加快外贸出口基地建设，积极开拓国际市场。深化与中国北方工业公司、中国保利集团等中央企业合作，实现借船出海。

民营企业是鞍山发展的活力所在。及时解决民营企业诉求，全面落实支持民营经济和中小微企业发展政策措施，着力化解企业融资难、融资贵问题，激发非公有制经济创造力。严格执行涉企收费清单制度，规范涉企收费行为。支持各类企业之间开展合资合作，在企业产权制度创新上实现突破。

鞍钢是鞍山未来经济发展的重要支撑。我们将全力支持鞍钢通过改革妥善解决历史遗留问题，切实做好鞍钢供水、供气、供暖和物业管理移交地方工作。支持鞍钢集团矿业公司老区改造、矿山三期开发和非钢产业发展。促进鞍钢、中冶等中省直企业与地方深度融合，共赢发展。

牢固树立投资环境就是生产力、竞争力的理念，努力打造良好的营商环境。继续实施市级领导包保项目责任制，切实解决事关企业发展、项目建设的难事急事，打造良好政务环境。全面实行项目代办制，打造优质服务环境。坚持公平公正、公开透明原则，摒弃重政府、轻市场，重速度、轻质量，重审批、轻服务，重国有、轻民营，重招商、轻营商的思维定势和管理方式，减少政府对企业和项目的行政干预，打造宽松有序的市场环境。

（二）大力推进改革

简政放权是改革的“先手棋”。继续巩固简政放权工作成果，确保已经下放的权力承接落实到位，解决好“最后一公里”问题。按照协同监管、优化服务的原则，规范事中事后监管。建立全地区网上审批系统和市级公共资源交易全流程电子化。深化开发园区体制机制创新，释放开发园区的发展活力、市场竞争力、对全市经济的引领力。深化商事制度改革，推进工商注册便捷化。

国有企业改革是今年改革工作的“重头戏”。坚持分类施策、分步实施、稳步推进、保持稳定的原则，加大国有资产整合力度，组建国有资本投资运营公司，盘活国有资产存量，促进国有资产保值增值。整合有效资产，拓展融资渠道。大力发展混合所有制企业，在体制机制上增强企业发展活力。

落实国税地税征管体制改革各项任务。合理调配财政、国资、社保资源，探索建立养老保险风险基金管理办法。健全政府性债务风险管控制度，着力预防和化解债务风险。完善金融机构逾期贷款清收工作事前报告制度，防止出现区域性金融风险。全面完成公车改革。

继续推进负面清单改革试点，积极探索放开公共服务、资源环境、生态环保、基础设施等领域市场准入。发挥全省企业投资项目管理体制改革试点作用，进一步优化建设项目审批流程，精简前置审批要件。加强政府与社会资本合作，引导社会资本投资基础设施和公用事业项目。

以海城国家综合改革试点为契机，在新型城镇化、农村集体经营性建设用地入市、农村集体资产股份权能改革、农村产权流转交易、县城基础设施投融资等方面先行先试，及时总结经验，在全市推广改革成果。完成100万亩土地确权登记颁证，推进全市农村产权交易市场体系建设，促进农村土地承包经营权流转。积极培育新型农业经营主体，鼓励工商资本投资农业，发展现代种养业、农产品深加工业、流通业和农业社会化服务业。

（三）积极促进大众创业万众创新

创新是引领鞍山发展的第一动力。以建设高新区创新产业园、经济开发区创业产业园为重点，实现每个县（市）区都有一个创新创业服务平台。鼓励企业建立实体型技术创新平台。引导企业、社会资本参与投资建设孵化器。加快建设科技大市场。全年新增国家高新技术企业20家，新增省级以上创新平台和企业研发机构20家。

深入实施“人才强市”战略。继续举办“院士专家鞍山行”活动，积极引进两院院士、长江学者和研发团队，新增院士工作站3家。继续实施“千人兴业”计划，积极引进海内外领军人才和优秀专家来鞍创业。建立校企联合职业技能人才培训体系，培养一批实用型技能人才。

积极融入沈大国家自主创新示范区和沈大高新技术产业带。加快建设国家知识产权示范城市和国家科技成果转化服务示范基地。设立产业（创业）投资引导基金，组建区域性科技成果转化综合服务平台。推动“政产学研用”协同创新，促进企业、大学和科研机构战略合作，开展产业链技术创新，促进科技成果转化和技术转移。

（四）加速调整工业结构

大力发展先进装备制造业。推动冶金与油气田成套装备、工业自动化与输变电装备、高端铸锻件与机械基础零部件、专用工程机械与车辆等产业升级，推进福鞍大型铸锻基地、瑞丰海洋装备、辽宁忠大高铁车厢及机器人等项目建设。

提升原材料及深加工产业。大力推广流程制造技术，提升钢铁产业数字化水平。促进钢铁精深加工产业，推进神龙腾达镀锌板、辽宁银恒家电卷板等项目建设。加快菱镁产业升级，推进海鸣矿业高端镁砂等项目建设。做强化工产业，推进缘泰化工炼化一体等项目建设。

培育壮大电子信息产业。围绕激光科技、电子信息制造等产业，深化互联网应用，引导企业以互联网为媒介，发展个性化定制、众包设计等新型制造模式。

积极培育新材料产业。围绕粉体新材料、功能性新材料、建筑新材料、纳米新材料产业，发展磁性材料、储能材料、集成建筑构件、光触媒等产品，推进鞍钢重机粉体材料、海华生态离子膜等项目建设。

加速发展节能环保和新能源产业。围绕工业三废资源化利用，推广电机永磁涡流传动技术和装备、变频调速装置等技术，发展新型高效燃煤工业锅炉、余热利用技术和装备。围绕光伏发电、电池、清洁能源产业，推进鑫普材料动力电池等项目建设。

（五）加快发展现代服务业

加速壮大现代物流业，推进辽宁钢铁物流基地等物流园区建设。做强商务服务业，推进万贯国际工业品博览中心建设。大力发展电子商务，建成西柳电子商务产业园。做大做强东北商品交易中心等大宗商品交易平台。继续推进生产性服务业分立。加速推进鞍山数字娱乐电子竞技产业园建设。积极谋划“健康制造”产业。

着力建设产业金融体系，加大金融支持实体经济力度。推进鞍山银行股权转让，与华阳集团战略重组。建成鞍山银行大连分行。完成海台岫三家农村信用合作社改制。加速推进一工集团、合成集团重整上市。促进9户企业实现境内外上市及区域股权交易市场挂牌。继续引进外埠金融机构。新增金融机构本外币贷款200亿元。

把做大做强旅游产业作为结构调整的重要突破口。积极探索国有重点景区经营体制改革，逐步实现所有权、管理权与经营权分离。深度挖掘旅游资源文化内涵，加速推进千山风景区创建国家5A级旅游景区，依托玉佛寺、龙泉寺等发展宗教文化旅游。加快千山温泉旅游度假区、汤岗子温泉产业集聚区等重点项目建设。进一步完善旅游基础设施。拓展精品旅游路线，扩大平面媒体和网络平台旅游宣传覆盖面。全年接待国内外游客和旅游总收入均增长15%。

通过农业转移人口市民化，释放新增市民住房需求，化解房地产库存，促进住房供需基本平衡。继续实行房屋市场交易阶段性激励政策，加大住房公积金对居民购房的支持力度，办好各类房交会。

（六）大力发展现代农业和县域经济

提高农业综合生产能力，粮食总产量达到130万吨。提高设施农业水平，积极创建设施蔬菜标准化基地和省级现代农业示范区。提高农业机械化水平，实现综合机械化率提高到79%。提高农业科技化水平，推广优质水稻栽培、超高产紧凑型玉米栽培等新技术10项，引进试验示范新品种18项，培训农村科技带头人80人，新型职业农民2000人。

加快县域工业园区建设，推进北京利尔菱镁深加工、金山彩涂板、佳合鹏程碳酸钙细粉等项目。加快县域服务业集聚区建设，推进西柳现代物流园、辽河文化旅游区、大洋河风情小镇等项目。加快县域农高区建设，推进海城三星花卉种苗繁育、台安安井速冻食品深加工二期、岫岩聚龙股份野生猕猴桃及深加工基地等项目。全年实现县域经济增速不低于全市平均水平。

全力实施扶贫攻坚计划。完善专项扶贫、行业扶贫、社会扶贫“三位一体”大格局，全面实施“六五五工程”。推进就业安置、移民搬迁、教育帮扶、医疗救助、生态补偿、低保兜底等“六个一批”社会保障工程。推进交通设施、电力设施、水利设施、信息化设施、生态环境等五项基础设施建设工程。推进特色农业、主导产业园区、生态文化旅游产业、新型城镇化建设、宝玉石集聚区建设等五大县域经济发展工程。年内完成0.9万国家标准贫困人口脱贫任务。

（七）统筹推进城乡建设

坚持集约发展，推动城市发展由外延扩张式向内涵提升式转变，不断完善城市基础设施，着力提高城市宜居性。科学编制新一轮鞍山城市总体规划，推进“多规合一”。推进建设大道等道路建设和千山西路暗渠等管网改造。推进机场航站楼改扩建，拓展西南、西北地区航线。推进辽河油田鞍山加气站建设，形成南北气源双向供给格局。坚持“建管并重”，采取政府向社会购买服务方式，推进市政基础设施维修、园林绿化养护和公共环卫作业等市场化运营。

以新型城镇化建设为重点，推进城镇化和宜居乡村建设，打造一批中心镇、特色镇。大力推进产城融合，促进农村劳动力向城镇集聚，推进高新区争创国家产城融合试点。注重以人为核心，促进基本公共服务均等化，切实提高城镇居民生活质量。稳步推进汤岗子新城建设，突出项目牵动，做实产业，聚集人气，重点推进鞍山路西延跨线桥，沈海高速公路汤岗子出口等项目建设，以交通一体化带动产业一体化。

树立蓝天、青山、碧水、净土是我们发展的最大本钱和最优生产力、核心竞争力的观念，强化环境治理。把大气污染防治作为头等大事，开展雾霾治理攻坚战。全力推进蓝天工程，完成鞍钢耐火材料总公司3号回转窑除尘改造工程、鞍矿大孤山球团厂烟气脱硫工程等20个重点项目，开工建设城市北部热电联产项目，加快谋划南部集中供热项目，新建改造锅炉40台、管网85公里，全面淘汰建成区10吨以下燃煤锅炉。实施生态脆弱区荒山造林和低效林改造。加快汤岗子新城污水处理厂建设。推进循环经济产业园生活垃圾焚烧发电等项目建设。

（八）切实保障和改善民生

继续实施民生工程。确保市委、市政府确定的20件民生实事如期完成，加快解决群众反映强烈的烟草岗拥堵、高新区广场恢复、玉佛山绿道延伸、文化场所建设等热点难点问题。完善创业服务体系，加强技能培训和创业培训，实现以创业带动就业。完善社会保障体系，加速推进“五险合一”系统建设。城乡最低生活保障标准分别提高5%和10%。加快回迁房建设，确保建成7个回迁房项目，建筑面积123万平方米，回迁居民1.5万户，实现在外过渡户全部回迁安置。继续推进棚户区、城中村、城边村搬迁改造，全面实行货币化安置。实施城市小区维修改造2万户，农村危房改造3000户，改造城乡社区办公用房56个。延伸2条城市公交线路，采购新能源公交车400辆、改造公交车150辆。改造农村敬老院25所。

加快发展社会事业。坚持创城惠民，深入推进全国文明城市创建工作。新增公益性学前教育学位5000个，改造农村薄弱学校20所，创建国家义务教育均衡发展示范县区，推进省现代职业教育试验区和国家产业转型技术技能人才双元培育改革试点工作，加快建设市职教城创业服务中心和继续教育培训中心，支持辽宁科技大学发展和鞍山师范学院新校区建设。深化医药卫生体制改革，协调推进医疗、医药、医保“三医联动”，大力推进公立医院人事分配制度改革，实行县域公立医院药品和耗材统一采购，推进医疗保险支付方式改革，市公共卫生服务中心建成使用，积极打造“健康鞍山”。全面落实一对夫妇可生育两个孩子政策，促进人口合理增长。推广医养结合养老服务模式。实施文化惠民工程，完善公共文化服务体系，推进市图书馆、群众艺术馆改造，增设自助图书馆。广泛开展全民健身运动，新增和维修城乡健身器材7000件，举办国际马拉松等重大体育赛事。继续做好双拥模范城创建工作，积极配合部队编制体制改革，开创军民融合发展新局面。

加速建设立体化社会治安防控体系，推进社会信息数据和视频图像资源整合，强化平安鞍山建设。健全生产安全隐患排查、治理和监管机制，严防发生重特大安全事故。建设市食品检验监测中心，加强食品药品安全监管，打造食品安全放心城市。畅通百姓诉求渠道，逐步化解历史遗留问题，维护人民群众合法权益，保持社会和谐稳定。

进一步做好广播电视、新闻出版、妇女儿童、老龄、残联、民族、宗教、外事、侨务、对台、统计、史志、档案、气象、地震、人防、民兵预备役等工作。

（九）加强政府自身建设

建设法治政府。贯彻落实国务院《法治政府建设实施纲要》，全面推进依法行政。完善政府科学决策机制，建立健全重大行政决策终身责任追究。强化层级监督，完善行政复议体制机制和法治队伍建设。自觉接受党内监督、人大监督、民主监督、司法监督，广泛接受社会各界监督。

建设诚信政府。政府要取信于民。政府作出的承诺，要及时兑现，不能说了不算。政府提供的信息，要真实准确，不能忽悠百姓。政府出台的政策，要言出法随，不能朝令夕改。

建设务实政府。坚持一切工作从实际出发，出实招、办实事、求实效，不求轰动效应，不做短期行为，不搞形象工程，要把一张好的蓝图绘到底。对于重点工作，要明确时间表和路线图，做到项目清、目标清、任务清、标准清、责任清，一抓到底，抓出成效。完善政府绩效考核机制，切实提高行政效能，对业绩突出的大力褒奖，对工作不力的约谈诫勉，对为官不为的严肃问责。

建设廉洁政府。严明党的政治纪律和政治规矩，坚持“三严三实”，讲诚信、懂规矩、守纪律，把纪律挺在前面，认真落实“一岗双责”。严格落实中央八项规定和省委十项规定，加大不作为、不担当问题整治力度，强化监督检查，促进作风转变。加强行政监察、审计监督、工作督查，加大行政问责力度，树立廉洁自律的新形象。

各位代表、同志们，“十三五”规划蓝图已经绘就，全面建成小康社会决胜期的号角已经吹响，让我们紧密团结在以习近平同志为总书记的党中央周围，在省委、省政府和市委的坚强领导下，在市人大、市政协的监督支持下，解放思想，开拓创新，真抓实干，为实现鞍山老工业基地全面振兴而努力奋斗！
